# On the Strong Composition Dependence of the Martensitic Transformation Temperature and Heat in Shape Memory Alloys

**DOI:** 10.3390/ma17164116

**Published:** 2024-08-20

**Authors:** Dezső L. Beke, Asmaa A. Azim

**Affiliations:** 1 Department of Solid State Physics, Doctoral School of Physics, University of Debrecen, P.O. Box 2, H-4010 Debrecen, Hungary; asmaa.abdelazim@science.unideb.hu; 2 Physics Department, Faculty of Science Ain Shams University, Abbassia, Cairo 11566, Egypt

**Keywords:** shape memory alloys, martensitic transformation, phonon softening, transformation temperature, heat and entropy, martensite start temperature

## Abstract

General derivation of the well-known Ren–Otsuka relationship, $\frac{1}{\alpha }\frac{dT_{o}}{dx}=-\frac{\alpha }{\beta }$ (where $T_{o}$, *x*, α and β(>0) are the transformation temperature and composition, as well as the composition and temperature coefficient of the critical shear constant, *c*′, respectively) for shape memory alloys, SMAs, is provided based on the similarity of interatomic potentials in the framework of dimensional analysis. A new dimensionless variable, $t_{o}\left(x\right)=\frac{T_{o}\left(x\right)}{T_{m}\left(x\right)}$, describing the phonon softening (where *T_m_* is the melting point) is introduced. The dimensionless values of the heat of transformation, Δ*H*, and entropy, Δ*S*, as well as the elastic constants *c*′, *c*_44_, and $A=\frac{c_{44}}{c^{\prime }}$ are universal functions of *t_o_*(*x*) and have the **same constant** values at *t_o_*(0) within sub-classes of host SMAs having the same type of crystal symmetry change during martensitic transformation. The ratio of $\frac{dt_{o}}{dx}$ and *α* has the same constant value for all members of a given sub-class, and relative increase in *c*′ with increasing composition should be compensated by the same decrease in *t_o_*. In the generalized Ren–Otsuka relationship, the anisotropy factor *A* appears instead of *c*′, and α as well as β are the differences between the corresponding coefficients for the *c*_44_ and *c*′ elastic constants. The obtained linear relationship between h and *t_o_* rationalizes the observed empirical linear relationships between the heat of transformation measured by differential scanning calorimetry (DSC) (*Q^A^*^⟶*M*^) and the martensite start temperature, *M_s_*.

## 1. Introduction

It is well known that the martensitic transformation temperature has strong composition dependence in shape memory alloys (SMAs) [1,2,3,4,5] and, for instance, 1 at% composition change can alter the transition temperature by about 100 K. In addition, the change of the content of defects (e.g., the change of concentration of vacancies by quench) can also have a similar effect [1]. The explanation of the above effect, even after the publication of a semi-quantitative derivation in [1], is still under discussion in the literature (see, e.g., [4,6,7,8,9,10,11,12,13]). In addition, it was found (see, e.g., Figure 5 in [3] and Figures 5b and 5d in [4]) that the transformation heat was a common linear function of the martensite start temperature, *M_s_*, in some different SMAs. We will also discuss this relationship and will derive it. 

It was shown in [1], from a Landau-type model of first-order phase transformations, that the martensitic transformation (MT) occurs at a critical elastic (basal plane shear) constant, *c*′ (=(*c*_11_ − *c*_12_)/2) and the value of it is constant at the transformation temperature, *T_o_*. Considering that the elastic constant has strong composition, *x*, and temperature dependence, it was concluded in [1] that the constancy of *c*′ at *T_o_* demands that the transformation temperature must exhibit an opposite effect, i.e., if *c*′ increases, *T_o_* should decrease with increasing composition. Thus, they arrived at the requirement

(1) $$c^{\prime }\left(x,T\right)=const.,\;at\;T=T_{o}.$$

Assuming that the composition and temperature dependence of *c*′ can be expressed as

$$c^{\prime }\left(x,T\right)=c_{o}^{\prime }\left(1+\alpha ∆x\right)\left(1+\beta ∆T\right),$$

where $c_{o}^{\prime }$ is the value of *c*′ at *T_o_*,

(2) $$\alpha =\frac{1}{c^{\prime }}\frac{dc^{\prime }}{dx}\;\mathrm{and}\;\beta =\frac{1}{c^{\prime }}\frac{dc^{\prime }}{dT},$$

the following relationship was obtained:

(3) $$\frac{∆T_{o}}{∆x}=\frac{dT_{o}}{dx}=-\frac{\alpha }{\beta },$$

or, using the definition of *α*,

(4) $$\frac{dT_{o}}{dc^{\prime }}=-\frac{1}{\beta C^{\prime }}.$$

It was also shown in [3] that, also taking into account that, typically, $\beta =\frac{4\%}{100K}=4\times 10^{-4}\frac{1}{K}$ [1,2,3,5,13] and $\alpha =4-10\frac{\%}{at\%}=\left(4-10\right)$ [1,11], $\frac{dT_{o}}{dx}$ has a negative sign and can be given as

(5) $$\frac{dT_{o}}{dx}=-\left(100-250\right)\frac{K}{at\%}.$$

This means that the transformation temperature is strongly affected by even a small change in composition or by quench. Investigating the validity of predictions (3) and (4) in [1], experimental data obtained in Cu-based shape memory alloys [2,3] as well as in Ti_50_Ni_30_Cu_20_ alloys [11] were used, implicitly suggesting that (3) and (4) can be general predictions for all SMAs, although according to their derivation *c*′ and $\frac{dT_{o}}{dx}$ can have different constant values at *T_o_*(0) in different alloy systems. Experimental data supported this expectation: the values of *c*′ and the $\frac{dT_{o}}{dx}$ slopes were slightly but definitely different in different SMAs [2,3,7,14,15,16,17]. For instance, it was shown in [14] that the value of *c*′ at *T_o_* was 30% smaller in *Ti*_50_*Ni*_30_*Cu*_20_ alloy than in binary NiTi. Furthermore, it was demonstrated that in Ni_2_MnGa $\frac{dT_{o}}{dx}$ was even positive (and α was negative) [7,15] and, e.g., the value of $\frac{dT_{o}}{dx}$ in TiPd-based shape memory alloys [17] with different alloying elements varied by about a factor of four (i.e., it changed between −15 K/at% and −60K/at%) by changing the type of the third alloying element. Since the softening of the corresponding elastic moduli is a key characteristic for martensitic transformations in SMAs, and not only *c*′, but *c*_44_ (belonging to non-basal plane shear) can also show softening, the above Ren–Otsuka relationships are expected to be valid only if *c*′ has phonon-softening behavior and *c*_44_ is practically independent of the temperature [11,12,13,18]. This latter assumption is, in a good approximation, valid for Cu-based alloys [5,18] or for Ti_50_Ni_30_Cu_20_ [14] but, e.g., in binary NiTi, both above moduli have phonon-softening-related temperature and composition dependence [11,12,13,18] and it was concluded (see, e.g., [14]) that the transformation temperature is more sensitive to the variation of *c*_44_ than to that of *c*′.

From the state-of-the-art, summarized above, it is clear that there is a need to generalize the semi-quantitative derivation of (3) and to get a definite answer on the following question: whether the predicted constants at *T_o_* are different in different alloys or there exists a classification rule providing groups of host SMAs, within which they are the same.

In this paper, we provide a different, general derivation of relationships of type (3) and (4), based on the law of corresponding states (LCSs) for metals with phonon softening. It will be shown that the general forms of (3) or (4), which contain the composition and temperature dependence of both *c*′ and *c*_44_, are just the consequence of the similarity of interatomic potentials [19]. For the derivation of it, one can avoid the use of phrasing like “critical value at the transition temperature” [1,4,5], or “criticality of the austenite”, which can be typical formulation for second-order phase transformations [20]. This is in line with the Ren–Otsuka approach, in which also no complete mode softening (if e.g., *c*′ ⟶ 0) is required [18] for first order phase transformations. In addition, since the explanation of the strong composition dependence of the heat of transformation, Δ*H*, and the linear relationship between Δ*H* and *M_s_* are still the question under debate [3,4,6,7,8,21] (e.g., in [21], it was concluded that composition dependence of Δ*H* “remains to be rationalized”), these will also be discussed. 

The organization of the paper is as follows. Basic relationships for the dependence of the transformation heat (∆H), entropy (ΔS), the shear constants *c*′, *c*_44_, and the anisotropy constant, $A=\frac{c_{44}}{c^{\prime }}$ on $t_{o}=\frac{T_{o}}{T_{m}}$ ($T_{m}$ is the meting point) are given in Section 2. The validity of the derived linearized relationships between $h=\frac{∆H}{kT_{m}}$ and $t_{o}$ is demonstrated on the examples of binary NiTi alloy (where the concentration, *x*, denotes the deviation from the stoichiometric (50/50 at%) composition on the Ni-rich side), in Cu_74.08_Al_13.13_Be_2.79_ alloys (where x shows the increase in the Be content from *x_Be_* = 2.79 at%), in Ti_50−x_Ni_40+x_Cu_10_ (where x changes between 0 at% and 1.2 at%) as well as in Ni_2_MnGa alloys (where x is the Ni excess in at% from the stoichiometric composition). In Section 3, our predictions will be compared with other experimental data. Section 4 contains the conclusions.

## 2. Derivation of the Basic Relationships

### 2.1. Law of Corresponding States for Phonon-Softening Systems

The LCS is the consequence of similarity of interatomic potentials [19], which can be written, in general, as:

Φ = εf(**r**_1_/**r**_o_, … **r**_N_/**r**_o_), (6)

where **r**_i_ is the position vector of the particle *i* (N is the number of atoms). Its form along a given direction can be represented by a periodic function with a period of *a_o_* and with an energy parameter of ε = f_max_ − f_min_ (e.g., similar to a sinus-type function with wavelength *λ* = *a_o_* and amplitude $A=\frac{\varepsilon }{2}$, where *a_o_* is the nearest neighbor distance). This shape is similar for all solids of the same bonding type (e.g., for metals and metallic alloys) and crystal structure, forming so-called similarity classes, i.e., *f* is the same function of its arguments within a similarity class.

In order to derive useful relationships between different physical quantities, one has to start from the fundamental theorem of dimensional analysis (“Pi theorem”) [22]. This is based on the dimensional homogeneity. It is well known that, in physics, there are fundamental and derived quantities. The fundamental quantities are dimensionally independent, and their number, *n*, is finite. It is easy to show that, e.g., the mass, *m*, the energy, *ε*, the length, *a_o_*, and the Boltzmann constant, *k*, can be taken as dimensionally independent quantities (the physical dimension of none of them can be combined from the others), forming the basis of the dimensional analysis [19,22]. According to the fundamental theorem, if *Q* denotes a (derived) physical quantity, then it can be given in the following form [19]:

Q = *m^a^ε^b^a_o_^c^k^d^*q(q_1_, …, q_g−n_). (7)

Here, q and its variables q_1_,…, q_g−n_ are dimensionless. This also means that q = Q/*m^a^ε^b^a_o_^c^k^d^* is dimensionless, i.e., the exponents *a*, *b*, *c*, and *d* should be chosen in such a way that the above dimensional combination of the fundamental quantities should give the dimension of Q. Furthermore, the number of independent dimensionless variables q_i_, in principle, is equal to *g* − *n*, where *g* is the number of variables present in the physically meaningful equation under investigation [22]. It can be shown that, neglecting quantum effects, and considering macroscopic (thermodynamic) quantities, in most of the cases, the only plausible variables are the dimensionless pressure and temperature [19,23,24]:

Q = *m^a^ε^b^ a_o_^c^k^d^*q(t, p), (8)

where the dimensionless temperature and pressure are given by t = *kT/ε* and *p* = *pa^3^/ε*. Furthermore, in accordance with Equation (6), *q* should be the same function for all members of the similarity class in question. It was shown that, using scaling parameters *kT_m_* (∝ε), *Ω* ($\propto a_{o}^{3}$), *m* and *k*, the relationships derived from (8) provided nice agreement with experimental data at *p* ≅ 0 for most metals and alloys (see, for instance, relationships for the diffusion and point defect properties [19,23,24,25]), even for binary alloys using composition-dependent melting points, *T_m_*(*x*), molar volume, *Ω*(*x*), and mass *m*(*x*). As it follows from (8), for the activation energy of diffusion at *p* ≅ 0

(9) $$\frac{Q_{D}}{T_{m}}=const.,$$

which is the well-known rule of thumb for self-diffusion in normal metals [23,25] (*Q_D_* is independent of *T*, according to the well-known Arrhenius-type *T-*dependence of the diffusion coefficient). On the other hand, for bcc metals showing phonon-softening behavior in the form of a curved Arrhenius function (anomalous behavior), introduction of one new dimensionless parameter, *ξ*, in the argument of *Q_D_* was needed [23,26]. According to [26,27], the curved Arrhenius plot can be described by the following temperature dependence of *Q_D_*:

(10) $$\frac{Q_{D}}{T_{m}}={\left.\frac{Q_{D}}{T_{m}}\right|}_{n}\left(1-\frac{T_{o}^{\prime }/T_{m}}{T/T_{m}}\right),$$

where ${\left.\frac{Q_{D}}{T_{m}}\right|}_{n}$ is the constant obtained in normal metals and $T_{o}^{\prime }$ was called, in [26], “hypothetical critical temperature”. In addition, it was shown in [26] that at a fixed (low) temperature the $\frac{Q_{D}}{T_{m}}$ ratio showed a linear dependence on the phonon-softening parameter, $\xi ={\left|\frac{v_{T}}{v_{L}}\right|}_{<111>}$, where *ξ* is the ratio of the transversal and longitudinal sound velocities along the <111> direction, i.e., $\xi \sim T_{o}^{\prime }$.

Since phonon softening is a key characteristic for martensitic transformations in SMAs, let us also formally introduce this parameter, denoted by ξ too, for shape memory alloys. Thus, we will use (8) in the form

(11) $$Q=m^{a}{\left(kT_{m}\right)}^{b}\Omega^{c}k^{d}q\left(t,\xi \right)$$

at atmospheric pressure ($t=\frac{T}{T_{m}}$, $p_{r}≅0$). Accordingly, the equilibrium transformation temperature, *T_o_*, can be given as

(12) $$T_{o}=T_{m}\vartheta \left(t_{o},\xi \right),$$

i.e.,

(13) $$t_{o}=\vartheta \left(t_{o},\xi \right).$$

Equation (13) means that a universal relationship should exist between ξ and *t_o_*. Thus, $\frac{T_{o}}{T_{m}}$ can be considered a good measure of *ξ* at $t=t_{o}$ and, in the following, *ξ* will be replaced by $t_{o}=\frac{T_{o}}{T_{m}}$. It is well known that *T_o_* has a strong composition dependence and thus *t_o_* should also have similar behavior.

### 2.2. Dependence of the Reduced Transformation Heat and Entropy on the Transformation Temperature

Let us first consider, in general, the *t_o_*-dependence of the heat of transformation, $h=\frac{∆H}{kT_{m}}$, and transformation entropy

(14) $$\frac{∆H}{{kT}_{m}}=h\left(t,t_{o}\right)≅h\left(t_{o}\right),$$

and

(15) $$\frac{∆S}{k}=s\left(t,t_{o}\right)≅s\left(t_{o}\right).$$

Here, we considered that ∆H and ΔS are usually independent of the temperature, *t.* Equation (14) means that *h* should depend universally on *t_o_* only and should have the same constant value at *t_o_*(0). Consequently, for instance, the $\eta_{h}=\frac{1}{h}\frac{dh}{dt_{o}}$ derivative should also have the same constant value for all SMAs at *t_o_*(0).

**Figure 1:**
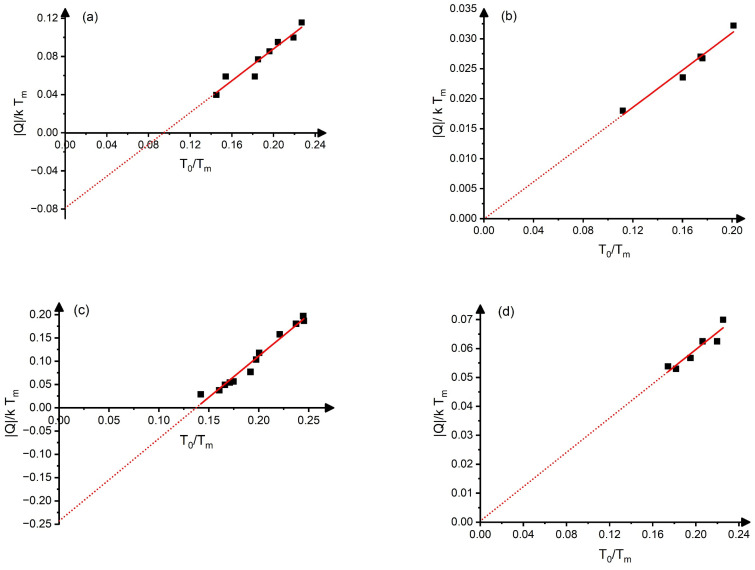
Graphs showing: $\frac{\left|Q\right|}{kT_{m}}$ versus $\frac{T_{o}}{T_{m}}$ in binary NiTi (**a**), in CuAlBe (**b**), in Ni_2_MnGa alloys (**c**), and in Ti_50−x_Ni_40+x_Cu_10_ (**d**) (on the basis of data published in [3,4,15,21], respectively (see also the text). The slopes, are 0.83, 0.16, 1.77, and 0.30, respectively. Blank squares are the experimental points and the dotted line is the extrapolation of the straight line fitted.

Thus, for shape memory alloys, we can plot $\frac{\left|∆H\right|}{{kT}_{m}}$ versus $t_{o}=\frac{T_{o}}{T_{m}}$, for NiTi (from [21], with $T_{m}≅1583\;K$), CuAlBe alloys (from [3], with $T_{m}≅1353\;K$), and Ni_2_MnGa (from [15], with $T_{m}≅1403\;K$), as well as for Ti_50−x_Ni_40+x_Cu_10_ (from [4], with $T_{m}≅1550\;K$) as it is shown in Figure 1. (In these plots ${\left|Q\right|=-Q}^{A⟶M}≅∆H$ and $T_{o}≅\frac{M_{s}+A_{f}}{2}$ assumptions were implicitly assumed, where $Q^{A⟶M}(<0)$ is the transformation heat measured by DSC, and *M_s_* and *A_f_* are the martensite start and austenite finish temperatures, respectively). 

It can be seen that these are linear functions, and the slopes of $\frac{\left|Q\right|}{{kT}_{m}}$ versus $\frac{T_{o}}{T_{m}}$ are 0.83, 0.16, 1.77, and 0.30 for NiTi, CuAlBe, Ni_2_MnGa, and Ti_50−x_Ni_50+x_Cu_10_, respectively. Furthermore, $\eta_{h}\left(t_{o}\left(0\right)\right)=\frac{1}{h}\frac{dh}{dt_{o}}=\frac{T_{m}}{∆H}\frac{d\left(\frac{∆H}{T_{m}}\right)}{dt_{o}}=8.0$ for NiTi, 5.2 for the CuAlBe, 59 for Ni_2_MnGa, and 4.2 for Ti_50−x_Ni_40+x_Cu_10_ systems, respectively (see also Table 1).

From the results shown in Figure 1, it can be seen that the *h*(*t_o_*), in the investigated parameter ranges, can be well approximated by straight lines. The corresponding slopes, as well as the values of *η_h_* at *t_o_*(0) (see also Table 1), although it would be expected that *h* has the same dependence on *t_o_* for all SMAs, are characteristically different. The main difference between the above four alloys is that they have different types of symmetry change during the martensitic transformation: (*B*_2_(*bcc*)*/B19*′(*monclinic*), in NiTi, *B*_2_(*bcc*)*/18R*(*rombohedral*), in CuAlBe, *L2*_1_(*bcc*)*/tetragonal*, in Ni_2_MnGa (where the structure of the tetragonal phase can be complex, being non-modulated or modulated [7]), and *B*_2_(*bcc*)*/B19*(*orhorombic*) in Ti_50−x_Ni_40+x_Cu_10_, respectively. Thus, plausibly, we have to make a distinction between SMAs on the basis of the type of symmetry change during the martensitic transformation; groups of alloys having the same symmetry change form different similarity sub-classes.

The above classification is also supported by the following arguments since, by definition, the dimensionless transformation entropy is given by

(16) $$s=\frac{∆S}{k}=\frac{∆H}{{kT}_{o}}=\frac{∆H/T_{m}}{kt_{o}},$$

and the transformation entropy, as it is well known [3], is also different for different sub-classes, i.e., it depends on the structure (symmetry) of the martensite. Furthermore, the fact that $\frac{∆H}{{kT}_{m}}$ is approximately a *linear* function of $\frac{T_{o}}{T_{m}}$ would dictate that the reduced entropy, *s*, should be independent of *t_o_* (and thus from *x*) within a sub-class, and has different constant values for different sub-classes. In addition, the function *h*(*t_o_*) should go through the origin.

**Figure 2:**
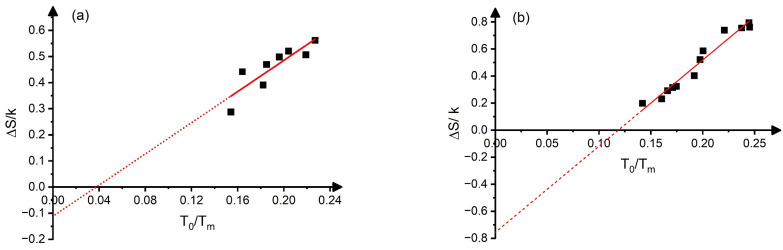
Graphs showing: $\frac{∆S}{k}$ versus $\frac{T_{o}}{T_{m}}$ in binary NiTi alloys [21] (**a**) and in Ni_2_MnGa alloys (**b**) [15]. The slopes, $\frac{d\frac{∆S}{k}}{dt_{o}}$, are 3.0 and 6.4, respectively (see also the text). Blank squares are the experimental points and the dotted line is the extrapolation of the straight line fitted.

It can be seen that it is quite well fulfilled for CuAlBe and Ti_50−x_Ni_40+x_Cu_10_, where the $\frac{\left|Q\right|}{kT_{m}}$ versus $\frac{T_{o}}{T_{m}}$ function goes through the origin, while the linear extrapolation of the fitted straight lines have definite intercept values for binary NiTi and binary Ni_2_MnGa alloys. In the case of CuAlBe and Ti_50−x_Ni_40+x_Cu_10_ alloys, it also means that the slopes in Figure 1 should be equal to the (constant) entropy, as calculated from the DSC data at *t_o_*(0): $s_{e}=\frac{∆S_{exp}}{k}=0.15$ and $\frac{∆S_{exp}}{k}=0.30$. It can be seen that the DSC data and the above slopes indeed agree very well (the slopes are 0.16 and 0.30, respectively). In addition, the experimental data of [3,4] confirm that *s* is indeed constant, i.e., independent of *t_o_*, in these alloys, and *s_e_* ≅ *s* (see also the Appendix A). In the case of NiTi and Ni_2_MnGa, the slopes provide different values than those of the experimental values of the entropies (calculated as ${∆S}_{exp}=\frac{\left|Q^{A⟶M}\right|+\left|Q^{M⟶A}\right|}{2T_{o}}$) at *t_o_*(0) (see also the data in Table 1). In addition, the $s_{e}=\frac{∆S_{exp}}{k}$ values show approximately a linear dependence on *t_o_*, as illustrated in Figure 2. Thus, both the linear dependence of $s_{e}$ and the different slopes of the $\frac{\left|Q\right|}{{kT}_{m}}$ versus *t_o_* plots from $s_{e}\left(t_{o}\left(0\right)\right)$ can be related to (i); the *s_e_*(*t_o_*) function is not constant, but the linear function of *t_o_* ($s_{e}=s_{eo}+\beta_{s}(t_{o}-t_{o}(0)$) and/or (ii), the approximations used when the $\frac{\left|Q\right|}{{kT}_{m}}$ versus $t_{o}$ is plotted instead of $\frac{∆H}{kT_{m}}$ versus $t_{o}$. As discussed in Appendix A, (i) has the dominating effect and $\frac{\left|Q\right|}{{kT}_{m}}$ is a quadratic function of *t_o_*, and the slope of the fitted linear relationship between $\frac{\left|Q\right|}{{kT}_{m}}$ and $t_{o}$, in a certain interval, is given approximately by $s_{e}\left(t_{o}\left(0\right)\right)+t_{o}\left(0\right)\beta_{s}=s_{eo}+t_{o}\left(0\right)\beta_{s}$.

Since *h* has composition dependence via its universal *t_o_*-dependence, a general relationship should exist between its composition derivative and $\frac{dt_{o}}{dx}$, as follows:

(17) $$\frac{1}{h}\frac{dh}{dx}=\frac{1}{h}\frac{dh}{dt_{o}}\frac{dt_{o}}{dx}=\eta_{h}\frac{dt_{o}}{dx}.$$

According to this, the universal constants $\eta_{h}$ can also be calculated from the ratio of the slopes, giving the composition dependence of *h* and *t_o_* (see also Table 1). 

It is worth emphasizing that the found approximately linear universal dependence of h on *t_o_* can also explain that, even if both ∆H and *T_o_* have non-linear dependence on *x*, ∆H versus *T_o_* can be linear (see, e.g., Figure 2a,b in [4] for binary NiTi, where the x-dependence of both *M_s_* and ∆H is non-linear, but ∆H versus *M_s_* is approximately a linear function. (Regarding the validity of assumption *M_s_* ≅ *T_o_*, see our comments in Section 3).

Before considering the corresponding expressions for the reduced (dimensionless) *c*′, *c*_44_, and *A*, it is worth recalling that besides the Boltzmann constant, the other three scaling parameters can also have composition dependence. But it can be easily accepted that the composition dependence of *m*, *Ω*, and *T_m_* is week as compared to the composition dependence of *T_o_* in SMAs. Indeed, in shape memory host alloys, the value of the atomic mass and volume is expected to have only a few percent change upon alloying. Furthermore, for the composition dependence of *T_m_*, we can take, as examples, the binary NiTi system as well as the CuAlBe systems, in which $\frac{1}{T_{o}}\frac{dT_{o}}{dx}≅-0.278\frac{1}{at\%}=-27.8,$ $\frac{1}{T_{m}}\frac{dT_{m}}{dx}=-\frac{1}{1583\;K}\frac{21\;K}{1\;at\%}=-0.013\frac{1}{at\%}=-1.13$ [21], $\frac{1}{T_{o}}\frac{dT_{o}}{dx}≅-21.7$, and $\frac{1}{T_{m}}\frac{dT_{m}}{dx}≅0$ [3]. Thus, for the sake of simplicity, in the following, only *T_o_* will be considered composition dependent.

### 2.3. Derivation of General Relationship for the Composition Dependence of the Transformation Temperature

Let us consider the elastic constant, *c*′, given in the following form:

(18) $$\;c^{\prime }=\frac{kT_{m}}{\Omega }\gamma_{c^{\prime }}\left(t,t_{o}(x)\right)$$

where $\gamma_{c^{\prime }}$ is a universal function of *t* and *t_o_*

(19) $$\gamma_{c^{\prime }}\left(t,t_{o}(x)\right)$$

and, at *t* = *t_o_*,

(20) $$\gamma_{c^{\prime }}\left({t=t}_{o},t_{o}(x)\right)=const.$$

According to Equations (18) and (19), the dimensionless *c*′ is a universal function of the reduced temperature, *t*, and the phonon-softening parameter, *t_o_*, within a given sub-class of SMAs. Equation (20) corresponds to Equation (1) in dimensionless form, and the *t_o_*-dependence is the consequence of the phonon softening, with an additional conclusion that the constant is expected to be the same within a sub-class but different in different sub-classes. From (19) (considering that $\frac{1}{\gamma_{c^{\prime }}}\frac{d\gamma_{c^{\prime }}}{dt_{o}}=\frac{1}{c^{\prime }}\frac{dc^{\prime }}{dt_{o}}$ and $\frac{1}{\gamma_{c^{\prime }}}\frac{\partial \gamma_{c^{\prime }}}{\partial x}=\frac{1}{c^{\prime }}\frac{dc^{\prime }}{dx}$ for composition-independent *T_m_* and *Ω*)

(21) $$\frac{1}{c^{\prime }}\frac{dc^{\prime }}{dt_{o}}=\frac{1}{c^{\prime }}\frac{dc^{\prime }}{dx}\frac{dx}{dt_{o}}.$$

According to (19), in the $\gamma_{c^{\prime }}=const.$ Condition, the constant value is the limit of the bilinear function of $\gamma_{c^{\prime }}\left(t,x\right)$ taken at *t* = *t_o_*(0). Introducing $t^{\prime }=t-t_{o}$ as a new variable in the vicinity of *t*_o_, the *t_o_*-dependence stems from the *t*’-dependence, and it is given by

(22) $$\frac{1}{c^{\prime }}\frac{dc^{\prime }}{dt_{o}}=\frac{1}{c^{\prime }}\frac{dc^{\prime }}{dt}\frac{dt}{dt^{\prime }}\frac{dt^{\prime }}{dt_{o}}=-\frac{1}{c^{\prime }}\frac{dc^{\prime }}{dt}.$$

Combination of (21) and (22) leads to

(23) $$\frac{dt_{o}}{dx}=-\frac{\alpha }{T_{m}\beta }.$$

This is just Equation (3) and, by the same arguments that were used in [1], Equation (4) is also valid, i.e.,

(24) $$\frac{dT_{o}}{dc^{\prime }}=-\frac{1}{\beta c^{\prime }}.$$

In addition, $\eta_{c^{\prime }}=\frac{1}{\gamma_{c^{\prime }}}\frac{d\gamma_{c^{\prime }}}{dt_{o}}=\frac{1}{c^{\prime }}\frac{dc^{\prime }}{dt_{o}}$ should have the same constant value at *t_o_*(0) within a sub-class, i.e.,

(25) $$\;\eta_{c^{\prime }}=\frac{1}{c^{\prime }}\frac{dc^{\prime }}{dt_{o}}=-\frac{1}{c^{\prime }}\frac{dc^{\prime }}{dt}=-\beta T_{m}.$$

Thus, the value of $\eta_{c^{\prime }}$ can be calculated from experimental data on *β* and *T_m_* (see also Table 3). In addition, from (23)

(26) $$\frac{1}{\alpha }\frac{dt_{o}}{dx}=-\frac{1}{\beta T_{m}}=\frac{1}{\eta_{c^{\prime }}},$$

and, since the right-hand side is the same negative constant value within a sub-class, we get that the ratio of slopes expressing the composition dependence of *t_o_* and *c*′ is constant, i.e., a relative increase in *c*′ with increasing composition should be compensated by the same relative decrease in *t_o_* (*β* > 0). This is a more definite statement than simply saying the lower α is, the higher $\frac{dT_{o}}{dx}$ is (see [1]).

As we summarized in the introduction, Equations (4) and thus (24) can be valid only if *c*′ shows phonon softening while *c*_44_ does not. The same results as above can be obtained by replacing *c*′ with *c*_44_ (with *β* now describing the phonon-softening-caused temperature dependence of *c*_44_); in this case the softening of *c*′ should be neglected. This conclusion is in line with the comments of [12], where it was mentioned that in (24) “… *c* can be either *c*_44_ or *c*′ …”.

Following the same procedure as above for *c*′ we can write for the dimensionless anisotropy factor, *A*

(27) $$A=\gamma_{A}\left(t,t_{o}\left(x\right)\right),$$

and

(28) $$\frac{T_{m}}{A}\frac{dA}{dT_{o}}=\eta_{A},$$

respectively; ($\eta_{A}=\frac{1}{A}\frac{dA}{dt_{o}}$) and $\eta_{A}$ and $A=\gamma_{A}$ are constants at $t_{o}\left(0\right).$ Thus, (see also (21) and (22)) finally,

$$\frac{dt_{o}}{dx}=-\frac{\alpha }{T_{m}\beta },$$

or

(29) $$\frac{dt_{o}}{\alpha dx}=-\frac{1}{T_{m}\beta }=const.$$

is obtained, where

(30) $$\alpha =\alpha_{c4}-\alpha_{c^{\prime }}=\frac{1}{c_{44}}\frac{dc_{44}}{dx}-\frac{1}{c^{\prime }}\frac{dc^{\prime }}{dx}$$

and

(31) $$\beta =\beta_{c4}-\beta_{c^{\prime }}=\frac{1}{c_{44}}\frac{dc_{44}}{dT}-\frac{1}{c^{\prime }}\frac{dc^{\prime }}{dT}.$$

Equation (29) is the generalized Ren–Otsuka relationship with generalized *α* and *β*. If the composition and temperature dependence of *c*_44_ can be neglected (or normal, i.e., it does not show phonon softening and its contribution to the martensitic transformation can be neglected) then one gets back the original Equation (3). For phonon-softening systems, the temperature coefficient contains two contributions. The positive one describes the direct phonon-softening contribution while the small, usually negligible, negative one is related to the usual (normal) anharmonicity-related softening of the crystal, and the first one dominates in the vicinity of *T_o_*. It can be noted that the anharmonicity-related temperature and composition dependence was also neglected in [1]. Furthermore, if both c′ and *c*_44_ have phonon softening in the above difference, the “normal” anharmonicity-related contributions approximately cancel out.

It is worth mentioning that the above approach, namely that the constancy of the anisotropy constant is the best starting point for finding a generalized relationship for the composition dependence of *T_o_*, can also be confirmed from the general form of the Landau expansion of the free energy, *F*, as used in [18]. This paper, instead of using only two strains (basal plane shear, *e*_1_, basal plane shuffle, *η* in Equation (1) of [1]), contained three ones: *e*_1_, *η*, and the $\left\{001\right\}<1\bar{1}0>$ non-basal plane shear, *e*_2_ (and c′, $\omega_{\eta }^{2}$, and *c*_44_ are the corresponding energy terms, respectively). Now it is easy to show that by minimizing *F* with respect to both *e*_2_ and *η* strains, one can get, besides condition (1) (and $\omega_{\eta }^{2}\left(T,x\right)=conts.$: see Equations (7a) and (7b) in [1]), that *c*_44_(*T*, *x*) has to also be constant at *T*=*T_o_.* Now, the ratio of Equation (1) and *c*_44_(*T*, *x*)=*const.* shows that *A* = *γ_A_* = *const.* at *t_o_*(0) (see Equation (27)). Of course, if there is no phonon softening in *c*_44_ (i.e., if its *t_o_*-dependence can be neglected) then only the phonon softening of *c*′ occurs, and the original Ren–Otsuka relationship can be approximately valid (like the case of the *Ti*_50*−x*_*Ni*_40*+x*_*Cu*_10_ or *CuAlBe* alloy with a large and increasing anisotropy by approaching *T_o_*). On the other hand, the *NiTi* alloys represent the other limit, when both *c*′ and *c*_44_ show phonon softening with a small value of *A* and a decreasing tendency of *A* with approaching *T_o_* [18] (i.e., $\eta_{A}>0$, see also Table 1 and the discussion below).

## 3. Comparison with Experimental Data

Some general features of the *t_o_*-dependence of the reduced characteristic quantities were already analyzed in the previous chapter, and it led to the conclusion that the host SMAs can be divided into sub-classes (having the same type of symmetry change during MT) within which the above quantities have the same constant values at t_o_(0). In this chapter, in addition to summarizing these, we also consider other SMAs to support the conclusions based on the data analyzed in Section 2.2. Furthermore, the reduced values of *c*′, *c*_44_, and *A* will be collected and compared with the data available in the literature. 

It has to be noted that, in many publications, the composition dependence of *T_o_* and *M_s_* is taken to be the same, and similarly to [1], we can also assume it here. *M_s_* can be given as $M_{s}=T_{o}-\frac{d_{o}+e_{o}}{-∆s_{c}}$, where *d_o_* and *e_o_* denote the first derivatives of the dissipative and elastic energies per unit volume, during the cooling process at the beginning of the transformation, and $∆s_{c}$ is the entropy change per unit volume during cooling [42]. The second term in *M_s_*, in fact, determines the dissipation and elastic energy accumulation, i.e., the above assumption means that we neglect the composition dependence of these terms, although, in a more refined treatment, this should be necessary to take into account, since, e.g., according to [8,9], the dissipative energy (the integral of *d_o_*) also shows a composition dependence. It is also worth mentioning that, in general, the x-dependence of *T_o_*(*x*) and the transformation heat, Δ*H*(*x*), are not strictly linear, but have a small downward curvature [8,21,30], but for the sake of simplicity, we neglect this moderate *x*-dependence of the slopes. Furthermore, it is also worth emphasizing that, in light of the results obtained in the previous chapter, it is indeed not expected that the values of the slopes $\frac{dt_{o}}{dx}=\frac{1}{T_{m}}\frac{dT_{o}}{dx}$ should have the same value even within a given sub-class, while, according to Equation (26) $\frac{1}{\alpha }\frac{dt_{o}}{dx}$ quantity should have the same value (see also Table 3 below).

Table 1 contains the values of those dimensionless constants at *t_o_*(0), which are predicted to be the same within a given subclass; *h*, *η_h_*, *s*, $\gamma_{c^{\prime }}$, $\gamma_{c44},$ *A* ≡ *γ_A_* and $\eta_{A}=\frac{1}{A}\frac{dA}{dt_{o}}=\frac{T_{m}}{A}\frac{dA}{dT}$. Table 2 contains the most important input parameters (*T_m_*, $\frac{T_{o}}{T_{m}}$, *c*′, *c*_44_, *β_c_*_′_, *β_c_*_44_, *α_c_*_′_, *α_c_*_44_, and $\frac{1}{T_{m}}\frac{dT_{o}}{dx}$) used in the calculation of the data given in Table 1. It has to be noted that most of the experimental data suffer from relatively large errors for the elastic parameters (typically between 15% and 25%), and there is a lack of reliable data, especially for the composition dependence of *c*′ and *c*_44_. Table 3 shows the estimated parameters related to the composition dependence of the transformation temperature.

**Figure 3:**
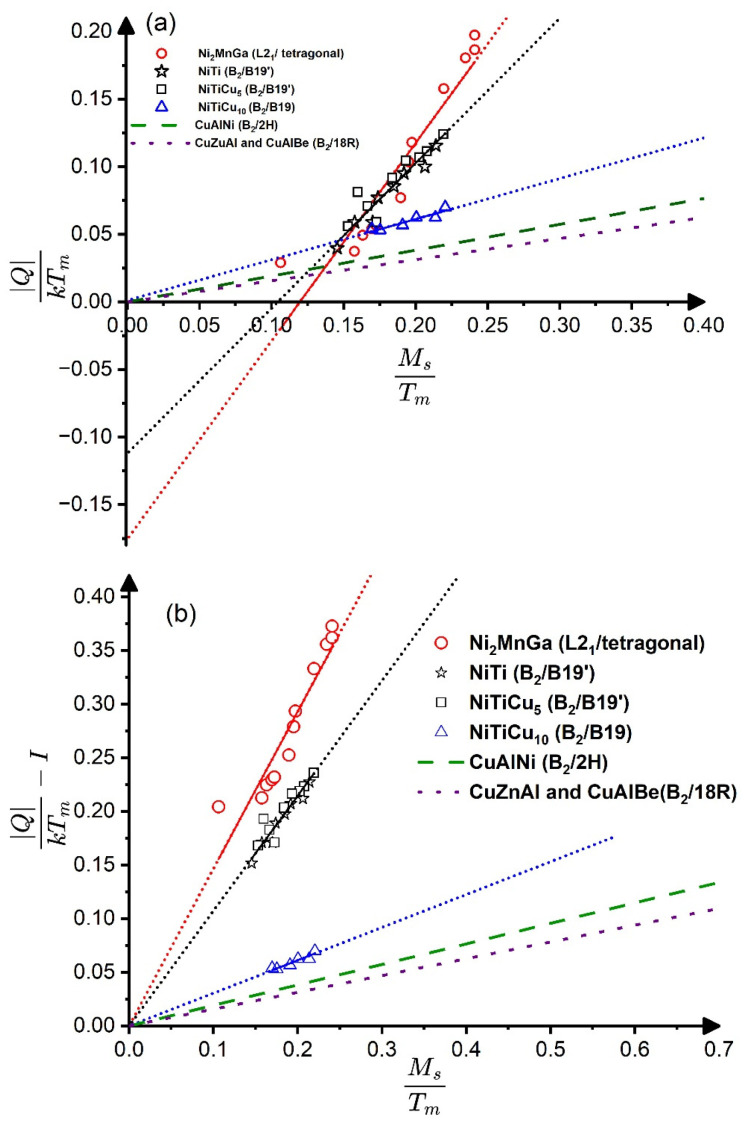
Graphs showing $\frac{\left|Q\right|}{kT_{m}}$ versus $\frac{M_{s}}{T_{m}}$ (**a**) as well as $\frac{\left|Q\right|}{kT_{m}}-I$ versus $\frac{M_{s}}{T_{m}}$ (**b**) plots (where *I* denotes the values of the intercepts in Figure 1 (see also the text). See also Figure 1 and Figure 2.

Table 1 contains the summary of the parameters predicted to be the same within the five sub-classes, represented by the NiTi, Ti_45−x_Ni_50+x_Cu_5_, Ti_50−x_Ni_40+x_Cu_10_, Ni_2_MnGa, Cu-Al-Be, CuZn, CuZnAl, and Cu_68_Al_28_Ni_4_ alloys. It can indeed be seen that the estimated values are characteristically different for the sub-classes. Furthermore, data for Cu-based alloys with B_2_/18R transformation (6th, 7th, and 8th rows) are rather similar, demonstrating that these quantities have the same constant values within a certain sub-class, as predicted. Regarding the CuAlNi (with B_2_/2H transformation), the constants are also not much different from the values of the above Cu-based alloys, suggesting that these two sub-classes behave similarly. On the other hand, the observations concluded in [3] support that the CuAlNi belongs to a different sub-class. In Figure 5 of [3], where the transformation heat ($\left|∆H^{A\to M}\right|$) was plotted versus the M_s_ temperature, the slopes of the straight lines were slightly but definitely different for CuAlNi (1.59 J/molK) from the common slope belonging to the fitted line on the data of CuAlBe and CuZnAl (1.30 J/molK; see also Figure 3 below). From the above slopes, $\eta_{h}=\frac{T_{m}}{∆H}\frac{d\left(∆H\right)}{dT_{o}}≅\frac{T_{m}}{∆H}\frac{d\left(∆H\right)}{dM_{s}}$, 5.2 and 4.8, respectively. It can be seen, as expected, that 5.2 is in a very good agreement with the value given in Table 1 for CuAlBe from Equation (17) (4.9) while, for CuAlNi, the agreement is still acceptable (from Equation (17) 3.9 was obtained). While this example also illustrates the experimental scatter (which is still in the range of the differences between 5.2 and 4.9 as well as 4.8 and 3.9), since the difference of the above slopes obtained on the basis of large number of experimental data collected in [3] was definite, one can confirm that the CuAlNi belongs to a different sub-class.

Interestingly, in Ni_2_MnGa, the signs of both α and $\frac{1}{T_{m}}\frac{dT_{o}}{dx}$ are even negative (see Table 2), and thus Equations (4) and (5) (as it was also mentioned in [7]) remain valid, since the sign of β is still the same as for other phonon-softening alloys (i.e., it is positive).

One additional comment supporting that bcc metals (and alloys) with phonon softening behave differently than the “normal” metals can be made. According to the above results, βT_m_ should be a universal constant (at t = 1, i.e., at T_m_) for all “normal” metals, while its value can be different for phonon-softening systems and, in addition, it should be different for different sub-classes of SMAs. The most salient result is that, indeed, β > 0, belonging to phonon-softening elastic constants in SMAs. On the other hand, it is negative, e.g., for Ag, Au, and Cu [43] (and βT_m_ is approximately constant for temperatures larger than the Debye temperature for all “normal” metals [43]: ~−0.56).

Table 3 contains the comparison of the predicted values of $\eta_{A}^{-1}=\frac{1}{{\alpha T}_{m}}\frac{dT_{o}}{dx}$ as well as $\eta_{A}^{-1}=-\frac{1}{\beta T_{m}}$ (columns 4th and 5th), as calculated at T_o_(0) from the experimental data given in Table 2. It can be seen that the signs in all cases are correct. *Note* (as it is also mentioned in the caption of Table 3) that for NiTi alloys, which are the most frequently used shape memory materials, only the generalized relationship provides the correct sign. Furthermore, as it can be seen from columns 5 and 6, the agreement between the value of $-\frac{\alpha }{\beta }$ and the experimental data for $\frac{dT_{o}}{dx}$ is also satisfactory, taking into account the uncertainties of the experimental values of α and β at present. As an example, we can mention the case of CuAlBe alloys. Here, the temperature dependence of the elastic constants is well known (as it is also shown in Table 2), and this slope does not change with the composition [2]. On the other hand, the composition dependence of c′ and c_44_ at room temperature and at the transformation temperature (see Figure 2 in [3]) is remarkably different (α_c′_ is about 10 at room temperature and about 0.7 at T_o_, or the composition dependence of A is given by $\eta_{A}$ = −9.3 as well as −1.0, respectively). In the Tables above, the room temperature value of α_c′_ was taken, since the reported value at T_o_ would lead to about an order of magnitude smaller value, although a value between 0.7 and 10 (and closer to 10) would lead to better agreement (α_c′_ ≅ 6.7 would lead to exact agreement between $-\frac{\alpha }{\beta }$ and the experimental $\frac{dT_{o}}{dx}$ value).

Finally, it is also worth adding that the constancy of $\frac{1}{{\alpha T}_{m}}\frac{dT_{o}}{dx}$ provides an explanation of the conjecture already proposed in 1988 by Verlinder and Delaey [44]: “the M_s_ temperatures of all the alloys can be correlated with an expression similar to that given for the composition dependence of c′ …” i.e., $\frac{1}{T_{m}}\frac{dT_{o}}{dx}\sim \alpha$.” In addition, they expressed that “similar calculations and conclusions as those presented in this paper for the two observations concerning the composition dependence of c′ and M_s_ could be made for the other alloy systems, providing the necessary experimental data are available”.

Finally, since, on the $\frac{\left|Q\right|}{kT_{m}}$ versus *t_o_* plots, the intercept *I* also depends on the position of the fitted *t_o_*-interval for systems in which the entropy has a linear *t_o_*-dependence, and thus $\frac{\left|Q\right|}{kT_{m}}$ versus *t_o_* is the quadratic function (see the Appendix A), it would be worthwhile to compile these plots into a common plot of $\frac{\left|Q\right|}{kT_{m}}-I$ versus *t_o_*. Furthermore, since the $\frac{\left|Q\right|}{kT_{m}}$ versus *M_s_* plots are more commonly used in the analysis of experimental data (see, e.g., [3,4]), Figure 3a,b shows the $\frac{\left|Q\right|}{kT_{m}}$ versus *t_o_* as well as $\frac{\left|Q\right|}{kT_{m}}-I$ versus $\frac{M_{s}}{T_{m}}$ plots. It can be seen in the compiled plots in Figure 3b that the only difference between the straight lines is that their slopes are different for different sub-classes of SMAs. Thus, this is a nice illustration of our prediction that the slopes of the $\frac{\left|Q\right|}{kT_{m}}$ versus *t_o_* plots should be different for SMAs with different symmetry changes during MTs. It is so, even if one takes into account that:

(i) In those systems where the entropy has an intrinsic *t_o_*-dependence, the slopes differ from the $s_{e}(t_{o}\left(0\right))=\frac{{∆S}_{exp}}{k}≅\frac{\left|Q\right|}{kT_{o}}$ values (shown in the fifth column of Table 1); and (ii) the slopes of $\frac{\left|Q\right|}{kT_{m}}-I$ versus $\frac{M_{s}}{T_{m}}$ are obviously slightly different from those of the $\frac{\left|Q\right|}{kT_{m}}-I$ versus *t_o_* plots (for instance the slopes are 1.11 as well 0.83 in NiTi or 0.160 and 0.156 in CuAlBe, respectively).

## 4. Conclusions

- It is shown that the application of the law of corresponding states for martensitic transformations of shape memory alloys with phonon softening requires the introduction of a new dimensionless phonon-softening parameter, which is proportional to $t_{o}=\frac{T_{o}}{T_{m}}$.
- Both the dimensionless heat and entropy of transformation ($h=\frac{∆H}{kT_{m}}$ and $s=\frac{∆S}{k}$) are universal functions of t_o_, and the composition dependences of them are determined by the composition dependence of t_o_ (or T_o_, since the composition dependence of T_m_ can be neglected).
- The slopes of the linearized h versus t_o_ plots were different for SMAs with different symmetry changes during martensitic transformation, forming sub-classes.
- Within a given sub-class, the normalized parameters, like the c′ elastic constant or the anisotropy constant ($\gamma =\frac{c^{\prime }\Omega }{kT_{m}}and\;A=\frac{c_{44}}{c^{\prime }}$), are the same constants at $\frac{T_{o}}{T_{m}}=t_{o}(0)$.
- From the above property of A, the generalized Ren–Otsuka relationship is obtained with generalized α and ββ parameters ($\alpha =\alpha_{c4}-\alpha_{c^{\prime }}=\frac{1}{c_{44}}\frac{dc_{44}}{dx}-\frac{1}{c^{\prime }}\frac{dc^{\prime }}{dx}$ as well as $\beta =\beta_{c4}-\beta_{c^{\prime }}=\frac{1}{c_{44}}\frac{dc_{44}}{dT}-\frac{1}{c^{\prime }}\frac{dc^{\prime }}{dT}$, respectively, where these are different from zero only for parameters showing phonon softening).
- It is shown that $\frac{1}{\alpha }\frac{dt_{o}}{dx}$ is the same constant within a given sub-class.
- The obtained a linear relationship between ∆H and T_o_ rationalizes the observed empirical linear relationships between the heat of transformation measured by DSC (Q^A⟶M^) and the martensite start temperature, M_s_.
- The latter two results will be important in understanding and classification of experimental results that will be obtained from new measurements on different SMAs.

## Figures and Tables

**Table 1:** Experimental dimensionless parameters at *t_o_*(0) in different sub-classes of SMAs (the references are given in the first column). For calculation of the dimensionless values, the melting points were estimated from the corresponding phase diagrams (see also Table 2) and we assumed that the atomic volume is the same for all alloys ($\Omega =\Omega_{NiTi}=8.4\times 10^{-6}\frac{m^{3}}{mol}$ [28]). In the third and fifth columns, values of $\eta_{h}$, calculated from the *t_o_*-dependence of *h* (from Equation (14) and Figure 1), as well as from Equation (21), respectively, are shown for comparison.

Sub-Class/Alloy		$\frac{∆H(0)}{{kT}_{m}(0)}$	$\eta_{h}(\frac{1}{h}\frac{dh}{dt_{o}})$	$\eta_{h}$ Equation (17)	$\frac{∆S_{exp}(0)}{k}$	*γ_c_*_′_	$\gamma_{c_{44}}$	A	$\eta_{A}=\frac{T_{m}}{A}\frac{dA}{dT}$
*B*_2_*/B*19′ *binary Ni*_50+*x*_*Ti*_50*−x*_ [4,5,14,21,29,30,31,32]		0.12	8.0	8.7	0.5	9.2	18	2.0	−2.9
*B*_2_*/B*19′ *Ti*_45*−x*_*Ni*_45+*x*_*Cu*_5_ (0 ≤ *x* ≤ +1.2 at%) [4]		0.12	5.3	4.9	0.56	-	-	-	-
B_2_/B19 *Ti*_50*-x*_*Ni*_40+*x*_*Cu*_10_ (0 ≤ *x* ≤ +1.2 at%) [4,29]		0.09	4.2	3.8	0.37	9.6	28	2.4	−5.3
*L2*_1_/tetragonal *Ni*_2+*x*_*Mn_x_Ga* [7,15,16,31,33,34]		0.03	59	60	0.20	9.4	80	8.4	−3.1
*B*_2_ */18R*	*CuAlBe* [2,3]	0.03	5.2	4.9	0.15	5.3	71.4	13.7	−1.0
	*CuZn* [5,14,35,36,37,38]	0.04	4.8	2.7	0.16	-	-	11	−0.8
	*CuZnAl* [2,3,35,36,37,38,39,40,41]	0.04	3.9	12	0.16	5.2	70.5	13.6	−1.0
B_2_/2H Cu_68_Al_28_Ni_4_ [2,3,31,40]		0.04	4.8	3.9	0.19	5.7	116	19	−2.85

**Table 2:** Experimental input parameters.

Sub-Class/Alloy		*T_m_* (*K*)	$\frac{T_{o}(0)}{T_{m}(0)}$	c′ (Gpa)	$c_{44}$ (GPa)	$\beta_{c^{\prime }}T_{m}$	$\beta_{c_{44}}T_{m}$	$\alpha_{c^{\prime }}$	$\alpha_{c^{\prime }}$	$\frac{1}{T_{m}}\frac{dT_{o}}{dx}$	$\frac{1}{h}\frac{dh}{dx}$
*B*_2_*/B*19′ *binary Ni*_50+*x*_*Ti*_50*−x*_ [4,5,14,21,29,30,31,32]		1583	0.23	14.4	28.6	2.5	5.4	−4	10	−6.3	−55
*B*_2_*/B*19′ *Ti*_45*−x*_*Ni*_50+*x*_*Cu*_5_ (−2 ≤ *x* ≤ +2) [4]		1583	0.26	-	-	-	-	-	-	−45	−4.3
B_2_/B19 *Ti*_50*−x*_*Ni*_40+*x*_*Cu*_10_ (0 ≤ *x* ≤ +1.2 at%) [4,14,27,29]		1550	0.22	14.5	34.53	3.9	−1.4	-	-	−62	−2.0
*L2*_1_/tetragonal *Ni*_2+*x*_*Mn_x_Ga* [7,15,16,31,33,34]		1403	0.15	12.8	107	3.1	~0	−15 *	~0 *	1.9	115
*B*_2_*/*18*R*	*CuAlBe* [2,3]	1353	0.20	7.0	95	0.46	−0.52	10	~0	−10.8	−53
	*Cu*_1*−x*_*Zn_x_* (0.38 ≤ *x* ≤ 0.50) [5,14,35,36,37,38]	1048	0.22	9.0	82	0.34	−0.46	6.5 **	~0	−6.1	−17
	*CuZnAl* [2,3,35,36,37,38,39,40,41]	1210	0.17	6.2	86	0.52	−0.48	3.5	~0	−6.1	−75
B_2_/2H Cu_68_Al_28_Ni_4_ [2,3,35,40]		1353	0.18	7.4	140	0.65	−2.2	4.5	~0	−7.4	−29

CuAlNi data for elastic constants and their T-dependence are averages of those published in [31,37], which deviate from the given average values by about ±18%. It is worth mentioning that in a recent paper [31] the temperature dependence of c′ and c_44_ was investigated in the very near vicinity of T_o_ in NiTi, Ni_2_MnGa, and CuAlNi and, except for the result for NiTi, their data provides about an order of magnitude larger values for β_c′_ than those given in Table 1 above. Data with upper index * are from theoretical papers also cited in the first column. Data for α_c′_ indexed by ** are estimated from the empirical relationship proposed by Veringen and Delaey [38] (see also [41]).

**Table 3:** Estimated parameters related to the composition dependence of the transformation temperature. The last column shows the experimental values of $\frac{dT_{o}}{dx}$ for comparison. Since, in Table 2, except for the binary NiTi alloy, in all cases, $\beta_{c_{44}}<0$ (i.e., *c*_44_ did not show phonon softening) the original Ren–Otsuka relationship (Equation (3)) with $\beta_{c^{\prime }}$ and $\alpha_{c^{\prime }}$ was used. For NiTi, the generalized relationship (Equation (29)) with $\beta =\beta_{c4}-\beta_{c^{\prime }}$ and $\alpha =\alpha_{c4}-\alpha_{c^{\prime }}$ was used ((Equation (3) would even lead to a positive value for $-\frac{\alpha }{\beta }$, i.e., the predicted value for $\frac{dT_{o}}{dx}$ would be wrong: $-\frac{\alpha }{\beta }=2533$).

Sub-Class/Alloy		$\beta T_{m}$	α	$-\frac{1}{\beta T_{m}}$	$\frac{1}{\alpha }\frac{dt_{o}}{dx}$	$-\frac{\alpha }{\beta }$	$\frac{dT_{o}}{dx}$
*ÍB_2_/B*19′ *binary Ni*_50+*x*_*Ti*_50−*x*_		2.9	14	−0.35	−0.45	−7642	−9973
*L2*_1_/tetragonal *Ni*_2+*x*_*Mn_x_Ga*		3.1	−15	−0.32	−0.13	6788	2666
*B*_2_/18*R*	*CuAlBe*	0.62	10	−1.6	−1.1	−21,823	−14,612
	*CuZn*	0.34	6.5	−2.9	−0.94	−8552	−6393
	*CuZnAl*	0.52	3.5	−1.9	−1.7	−8144	−7381
B_2_/2H Cu_68_Al_28_Ni_4_		0.65	4.5	−1.5	−1.6	−9370	−10,012

**Figure A1:**
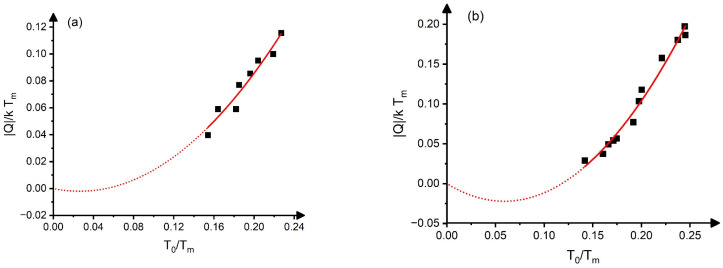
Graphs showing $\frac{\left|Q\right|}{kT_{m}}$ versus *t_o_* plots for NiTi (**a**) and Ni_2_MnGa (**b**). The fitted curves show the fit with Equation (A9) and both fitting parameters are in very good agreement with data given in Table A1 (obtained from the linear of *s_e_* versus *t_o_*): $s_{eo}-\beta_{s}t_{oo}=-0.15$ and $s_{eo}-\beta_{s}t_{oo}=-0.75$ as well as $\beta_{s}=2.9$ and $\beta_{s}=6.4$, for NiTi and Ni_2_MnGa systems, respectively. The same is valid for the positions of the minima. Blank squares are the experimental points and the dotted line demonstrate the fitted quadratic function.

## Data Availability

Data will be available upon request.

## Appendix A

The slope and intercept of $\frac{\left|Q\right|}{{kT}_{m}}$ versus *t_o_* plots.

The heats of transformation measured by a calorimeter for cooling as well as heating (*A*⟶*M*, and *M*⟶*A*, respectively) contain additional terms arising from elastic energy accumulation and dissipation losses (non-chemical energy terms) [3,29]:

(A1) $${\left|Q\right|=-Q}^{A⟶M}=-∆H^{A⟶M}-E^{A⟶M}-D^{A⟶M}=∆H-E-D,$$

and

(A2) $$Q^{M⟶A}=∆H^{M⟶A}-E^{A⟶M}+D^{A⟶M}=∆H-E+D.$$

Here, $∆H=-∆H^{A⟶M}=∆H^{M⟶A}>0$ is the transformation enthalpy and $E^{A⟶M}=-E^{M⟶A}=E>0$ and *D*(>0) denotes the elastic and dissipative energy, assuming that *D* is the same in both directions and that the same elastic energy is released during heating what was accumulated during cooling (which explains the negative sign in (A2)). Thus, using the Tong–Wayman approximation ($T_{o}≅\frac{M_{s}+A_{f}}{2}$), we can write from (A1),

(A3) $$\frac{\left|Q\right|}{kT_{m}}=\frac{∆H}{kT_{m}}-\frac{E+D}{kT_{m}}=st_{o}-\frac{E+D}{kT_{m}},$$

where the definition of the transformation entropy, $s=\frac{∆S}{k}=\frac{∆H}{{kT}_{o}}$, was also used. Furthermore, the experimental values of *s* are calculated from the relationship $s_{e}=\frac{{∆S}_{exp}}{k}≅\frac{\left|Q\right|}{kT_{o}}$ as

(A4) $$s_{e}=\frac{{∆S}_{exp}}{k}≅\frac{\left|Q\right|}{kT_{o}}≅s-\frac{E+D}{{kT}_{o}}.$$

Combination of (A3) and (A4) gives

(A5) $$\frac{\left|Q\right|}{kT_{m}}=s_{e}t_{o}.$$

Thus, for the slopes of $\frac{\left|Q\right|}{kT_{m}}$ versus *t_o_* as well as of *s_e_* versus *t_o_* we get

(A6) $$\frac{d(\frac{\left|Q\right|}{{kT}_{m}})}{dt_{o}}=s_{e}$$

and

(A7) $$\frac{ds_{e}}{dt_{o}}=0$$

if *s_e_* is constant.

We have seen, in the main text, that for CuAlBe and Ti_50−x_Ni_40+x_Cu_10_ alloys, *s_e_* was indeed independent of *t_o_*, and $\frac{\left|Q\right|}{kT_{m}}$ versus *t_o_* was linear and went through the origin and $s_{e}≅\frac{d(\frac{\left|Q\right|}{{kT}_{m}})}{dt_{o}}$. In addition, since $\frac{E+D}{kT_{m}}$ is typically not larger than about 10–20% of $\frac{∆H}{kT_{m}}$ and, using data from Table 1 in the main text $\frac{∆H}{kT_{m}}≅0.09$ in Ti_50−x_Ni_40+x_Cu_10_ as well as 0.03 in CuAlBe and thus $\frac{E+D}{kT_{m}}≅0.018-0.009$ as well as 0.002–0.001, respectively (while $s_{e}=0.3$ and 0.15, respectively). Thus, *s_e_* ≅ *s* also holds.

For the NiTi and Ni_2_MnGa systems, the condition (A7) did not fulfill, and Table A1 shows the corresponding values of the slopes and the intercepts of the $\frac{\left|Q\right|}{kT_{m}}$ versus *t_o_* as well as $s_{e}$ versus *t_o_* plots. In these alloys, there is a well-defined linear dependence of *s_e_* on *t_o_*, which can be attributed to other than vibrational contributions (which are fully related to the symmetry change during the martensitic transformation [3,15]) and, for instance, in [15], the above dependence was interpreted by magnetic contribution to *s_e_* in Ni_2_MnGa. Thus, we can write for such an intrinsic dependence:

(A8) $$s_{e}=s_{eo}+\beta_{s}\left(t_{o}-t_{oo}\right)=s_{eo}-\beta_{s}t_{oo}+\beta_{s}t_{o},$$

where $s_{eo}=s_{e}\left(t_{o}\left(0\right)\right)$ and $t_{oo}=t_{o}\left(0\right)$ are constants. Putting (A8) into (A5) we get

(A9) $$\frac{\left|Q\right|}{kT_{m}}=t_{o}\left[s_{eo}+\beta_{s}\left(t_{o}-t_{oo}\right)\right]=\left(s_{eo}-\beta_{s}t_{oo}\right)t_{o}+\beta_{s}t_{o}^{2},$$

and thus

(A10) $$\frac{d\frac{\left|Q\right|}{kT_{m}}}{dt_{o}}=s_{eo}-\beta_{s}t_{oo}+{2\beta }_{s}{\underline{t}}_{o}=s_{eo}+\beta_{s}\left(2{\underline{t}}_{o}-t_{oo}\right),$$

where ${\underline{t}}_{o}$ is the median value of the interval where the linear fit was made to the $\frac{\left|Q\right|}{kT_{m}}$ versus *t_o_* plot. It can be seen from (A9) that $\frac{\left|Q\right|}{kT_{m}}$ is a quadratic function of *t_o_*, as is illustrated in Figure A1, and the linear fit is made in the certain *t_o_* interval with *t_o_* median value (*t_o_* ≅ 0.20 and 0.21 for NiTi as well as Ni_2_MnGa, respectively). Thus, the intercepts of the linearized $\frac{\left|Q\right|}{kT_{m}}$ versus *t_o_* have no physical meaning (and depend on the position of the fitted range). In contrast, the slopes of the $\frac{\left|Q\right|}{kT_{m}}$ versus *t_o_* as well as $s_{e}$ versus *t_o_* plots are relevant parameters, and, as one can check it from the data collected in Table A1, they form a consistent set, in accordance with Equations (A8) and (A9). It can be noted that the position of the minimum in Figure A1 (form (A9)) is given by $t_{omin}=\frac{1}{2}\left(t_{oo}-\frac{s_{oo}}{\beta_{s}}\right)$, i.e., it is determined by the fitting parameters of the linear *s_e_* versus *t_o_* function.

**Table A1:** Slope and intercepts of the $\frac{\left|Q\right|}{kT_{m}}$ versus *t_o_* as well as $s_{e}$ versus *t_o_* plots for NiTi and Ni_2_MnGa alloys.

Alloy	$\mathrm{Slope}\;\mathrm{of}\;\frac{\left|Q\right|}{kT_{m}}$ versus *t_o_*	$\mathrm{Intercept}\;\mathrm{of}\;\frac{\left|Q\right|}{kT_{m}}$ versus *t_o_*	Slope of *s_e_* versus *t_o_*	Intercept of *s_e_* versus *t_o_*	$s_{eo}=≅\frac{\left|Q\right|}{kT_{o}}$	$t_{o}\left(0\right)=t_{oo}$	${\underline{t}}_{o}$
NiTi	0.83 ± 0.10	−0.08 ± 0.02	3.0 ± 0.7	−0.11 ± 0.13	0.50	0.23	0.20
Ni_2_MnGa	1.78 ± 0.10	−0.25 ± 0.02	6.4 ± 0.5	−1.1 ± 0.1	0.20	0.15	0.21
